# Under the Hood: Skeletal Muscle Determinants of Endurance Performance

**DOI:** 10.3389/fspor.2021.719434

**Published:** 2021-08-04

**Authors:** Stephan van der Zwaard, Franck Brocherie, Richard T. Jaspers

**Affiliations:** ^1^Department of Human Movement Sciences, Vrije Universiteit Amsterdam, Amsterdam Movement Sciences, Amsterdam, Netherlands; ^2^Laboratory for Myology, Department of Human Movement Sciences, Vrije Universiteit Amsterdam, Amsterdam Movement Sciences, Amsterdam, Netherlands; ^3^Leiden Institute of Advanced Computer Science, Leiden University, Leiden, Netherlands; ^4^Laboratory Sport, Expertise and Performance (EA 7370), French Institute of Sport (INSEP), Paris, France

**Keywords:** V̇O_2max_, training, metabolism, aerobic performance, muscle fiber type, oxygen transport, mitochondria, hypertrophy

## Abstract

In the past decades, researchers have extensively studied (elite) athletes' physiological responses to understand how to maximize their endurance performance. In endurance sports, whole-body measurements such as the maximal oxygen consumption, lactate threshold, and efficiency/economy play a key role in performance. Although these determinants are known to interact, it has also been demonstrated that athletes rarely excel in all three. The leading question is how athletes reach exceptional values in one or all of these determinants to optimize their endurance performance, and how such performance can be explained by (combinations of) underlying physiological determinants. In this review, we advance on Joyner and Coyle's conceptual framework of endurance performance, by integrating a meta-analysis of the interrelationships, and corresponding effect sizes between endurance performance and its key physiological determinants at the macroscopic (whole-body) and the microscopic level (muscle tissue, i.e., muscle fiber oxidative capacity, oxygen supply, muscle fiber size, and fiber type). Moreover, we discuss how these physiological determinants can be improved by training and what potential physiological challenges endurance athletes may face when trying to maximize their performance. This review highlights that integrative assessment of skeletal muscle determinants points toward efficient type-I fibers with a high mitochondrial oxidative capacity and strongly encourages well-adjusted capillarization and myoglobin concentrations to accommodate the required oxygen flux during endurance performance, especially in large muscle fibers. Optimisation of endurance performance requires careful design of training interventions that fine tune modulation of exercise intensity, frequency and duration, and particularly periodisation with respect to the skeletal muscle determinants.

## Introduction

Endurance athletes compete against each other in a race to finish first. Independently of the sport performed, these athletes aim to reach the finish line as quickly as possible, by producing the highest average velocity over the course of the event. Endurance competitions typically range from ~3 to 230 min within sports such as running, swimming, speed skating, skiing, and cycling (Riegel, 1981), excluding ultra-endurance competitions. Within such performance duration, world records highlight a distinct linear log-log relationship between finishing times and distance covered for many endurance sports (Lietzke, 1954; Riegel, 1981). The slope of these equations quantifies human endurance, the highest average velocity that is sustained over a given time period or the longest time a given velocity is sustained (Lietzke, 1954; Riegel, 1981; Billat et al., 1999). Later work also assessed the relationships between power and exercise duration, which presumably link more directly to the athlete's underlying physiology (Billat et al., 1999). Such power-duration profiles are widely applied in endurance sports (e.g., cycling) to determine endurance performance and the physical/physiological signature of an athlete (Pinot and Grappe, 2011; Sanders and Erp van, 2020). In a conceptual framework, Joyner and Coyle (2008) already described “potential” physiological determinants of endurance performance. In this review, we advance on this framework, now including a meta-analysis highlighting the (effect size of) interrelationships between endurance performance and key physiological determinants of endurance performance at the macroscopic and microscopic level. Moreover, we discuss how these physiological determinants can be improved by training. Note that this mini review focuses mainly on structural and functional muscle (fiber) characteristics rather than other relevant topics for endurance performance, such as pulmonary or circulatory physiology or nutrition.

## Whole-Body Determinants of Endurance Performance

Already in 1925, A.V. Hill recognized that fatigue was underlying the decline in velocity with increasing race distance (Hill, 1925). He speculated that performance was determined by physiological characteristics related to energetic costs, oxygen demands, and supply, and oxygen debt (or racing economy). Almost a century later, Joyner and Coyle (2008) highlighted the importance of the maximal oxygen uptake ($\dot{\text{V}}$O_2max_), lactate threshold, and efficiency/economy in endurance performance. The $\dot{\text{V}}$O_2max_ and lactate threshold interact to determine how long a given rate of aerobic and anaerobic metabolism can be sustained (i.e., performance $\dot{\text{V}}$O_2_) by an athlete, whereas efficiency determines the velocity or power that can be achieved with a given amount of energy consumption (Joyner and Coyle, 2008).

Elite endurance athletes may score exceptionally high on one (or several) of these three determinants. Upper limits for absolute $\dot{\text{V}}$O_2max_ have been reported in rowers and cross-country skiers (7.0–7.5 L·*min*^−1^ *in males and* 5.0−5.5 *L*·*min*^−1^ in females) and for relative $\dot{\text{V}}$O_2max_ in cyclists, runners, and cross-country skiers (up to ~90 *mL*·*kg*^−1^·*min*^−1^ *in males and* ~80 *mL*·*kg*^−1^·*min*^−1^ in females) (Haugen et al., 2018; van der Zwaard et al., 2018c). The (first) lactate threshold discriminates moderate- from heavy-intensity exercise and can be determined by gas exchange and/or blood lactate measurements (Poole et al., 2020). Typically, values approximate 75–85% of $\dot{\text{V}}$O_2max_ in athletes (Coyle et al., 1988; Seiler and Kjerland, 2006; Joyner and Coyle, 2008). Knowing that all Olympic endurance events are decided at intensities above 85% of $\dot{\text{V}}$O_2max_ (Joyner and Coyle, 2008), athletes benefit from being relatively fatigue resistant at high exercise intensities and in this context a high lactate threshold can be very advantageous. As for gross efficiency, values generally range ~20–23% in elite cyclists (Jeukendrup et al., 2003; Ettema and Lorås, 2009) and ~18–19% in elite rowers (Bourdin et al., 2004). In world-class cyclists, Lucía et al. (2002b) reported efficiency values up to ~24.5–28.1%, although possible methodological issues may be at play (Jeukendrup et al., 2003). Even in elite athletes, scoring high on all three determinants remains unusual, as illustrated by the inverse relationship between $\dot{\text{V}}$O_2max_ and gross efficiency (Lucía et al., 2002a). However, interestingly, a two-time Tour de France champion was able to display both a very high $\dot{\text{V}}$O_2peak_ (84 mL·kg^−1^·min^−1^) and gross efficiency (23.2%), together with a reasonably high lactate threshold (~81% of $\dot{\text{V}}$O_2peak_), which is likely contributing to his athletic success (Bell et al., 2017).

## The Overlooked Underlying Physiology: Skeletal Muscle Determinants of Endurance Performance

Discussing the physiology of champions, Joyner and Coyle (2008) already suggest that several anthropometrical, cardiac, and skeletal muscle characteristics underlie endurance performance and its whole-body determinants. In particular, skeletal muscular properties could explain differences in (whole-body determinants of) endurance performance, and have been almost exclusively derived from invasive muscle biopsies (Bergstrom, 1975). Although not commonly practiced with elite athletes, biopsies provide valuable and reproducible data on human muscle physiology using only a few 100 muscle cells (Ekblom, 2017), such as on muscle fiber type, cross-sectional area, mitochondrial volume density or function and capillarization, and can be safely obtained (Tarnopolsky et al., 2011). Yet, the interrelationships and corresponding effect sizes between these physiological determinants and endurance performance remain largely overlooked.

### Muscle Fiber Type

Muscle fiber type is important for endurance, and its characteristics have been reviewed in detail elsewhere (Schiaffino and Reggiani, 2011). Researchers in the 1970–80s extensively investigated muscle fiber type composition in athletes (Gollnick et al., 1972; Costill et al., 1976; Komi et al., 1977; Saltin et al., 1977; Hagerman and Staron, 1983; Tesch et al., 1984; Tesch and Karlsson, 1985; Yazvikov et al., 1988). They discovered that long-distance runners, rowers, swimmers, and kayakers predominantly had slow type-I muscle fibers (~70–80%), in greater proportion than long-distance speed skaters, middle-distance runners, skiers and cyclists (~50–60%), jumpers, and throwers (~40–50%), weight lifters, wrestlers, power lifters, and sprint speed skaters (~25–45%) or sprint runners (~25%). Recent literature confirmed that a champion sprint runner had only a limited proportion of type-I fibers (29%), but a high percentage of powerful type-II fibers (Trappe et al., 2015). Elite road and team pursuit cyclists likely have a higher proportion of type-I fibers than previously thought (~65–75%) (Sjøgaard, 1984; Coyle et al., 1991; Aagaard et al., 2011; van der Zwaard et al., 2018b), similar to that of other endurance athletes. Of note, distinct sport-dependent (e.g., kayaking vs. cycling vs. running) differences in muscle fiber composition have been reported between arm and leg muscles (Gollnick et al., 1972; Tesch and Karlsson, 1985), illustrating the sport-specific requirements and long-term training effects. Given the fact that high proportions of type-I fibers are suggested to display greater mechanical efficiency at common movement frequencies (i.e., 1–2Hz, e.g., cycling between 60 and 120 rpm) that are close to the type-I fiber's peak efficiency velocity (He et al., 2000; Joyner and Coyle, 2008), a higher percentage of type-I fibers would relate to a higher gross efficiency (Coyle et al., 1992; Horowitz et al., 1994), and therefore contributes to a higher endurance performance (Horowitz et al., 1994). Nowadays, non-invasive techniques such as ^1^H-MRS measurements of muscle carnosine content are also used to estimate type-I and II muscle typology, thereby confirming prior muscle biopsy-based findings across athletes (Baguet et al., 2011; Bex et al., 2017; Lievens et al., 2020, 2021).

### Muscle Fiber Cross-Sectional Area

**Figure 1:**
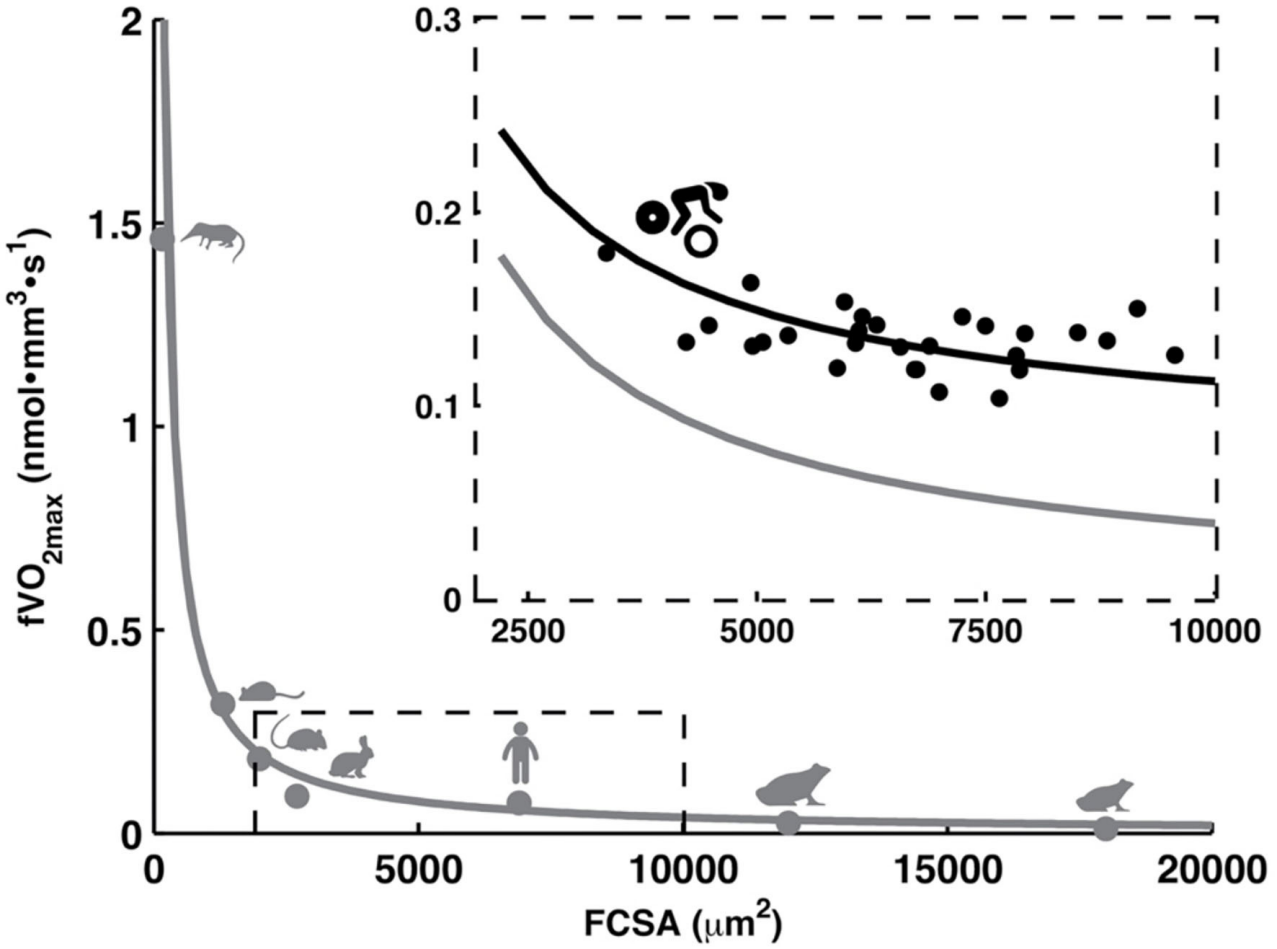
Muscle fiber size (FCSA) and muscle fiber oxidative capacity ($\dot{\text{V}}$O_2max_) are inversely related across animal species [*r* = −0.98, *P* < 0.001 (van der Laarse et al., 1998; van Wessel et al., 2010)] and in competitive cyclists [*r* = −0.50, *P* < 0.05 (van der Zwaard et al., 2018b)]. From left to right, animals include the shrew, mouse, rat, rabbit, human, African clawed frog (Xenopus laevis), and frog (Rana temporaria). Figure is created based on data from van der Laarse et al. (1998), van Wessel et al. (2010), van der Zwaard et al. (2018b).

Muscle fiber cross-sectional area (FCSA) contributes to the muscle's force production and is regulated by protein synthesis and degradation (van Wessel et al., 2010). In humans, FCSA typically ranges from 3,000 to 10,000 μm^2^ (Gollnick et al., 1972; Costill et al., 1976; Saltin et al., 1977; Sjøgaard, 1984; Tesch and Karlsson, 1985; Aagaard et al., 2011; van der Zwaard et al., 2018b) with generally smaller size in type-I compared to type-II fibers (Gollnick et al., 1972; Costill et al., 1976). Accordingly, endurance athletes report smaller values (~6,000 μm^2^) compared to track sprinters (~8,000 μm^2^) and weight/power lifters (~9,000 μm^2^). Yet, the relative muscle area that is occupied by type-I fibers is considerably larger in endurance compared to non-endurance athletes (Gollnick et al., 1972; Costill et al., 1976). Notably, in some elite cyclists, type-I FCSA may even be larger than FCSA of their type-II fibers (Sjøgaard, 1984). In this view, having large oxidative muscle fibers theoretically benefits endurance performance. In practice, however, FCSA appears negatively related to endurance performance (Coyle et al., 1988; Bishop et al., 2000) and lactate threshold (Bishop et al., 2000) in cyclists. The muscle fiber type-fiber size paradox may explain this through a profound inverse relationship between FCSA and muscle fiber oxidative capacity across animal species (van der Laarse et al., 1998; van Wessel et al., 2010) and in competitive cyclists (van der Zwaard et al., 2018b). This illustrates a trade-off between both traits, which makes it difficult to concurrently optimize FCSA and muscle fiber oxidative capacity (Figure 1).

### Mitochondrial Oxidative Capacity

Muscle fiber oxidative capacity resembles the mitochondria's ability to deliver ATP using oxygen, which provides the necessary energy to sustain prolonged exercise. Within the mitochondria, energy is delivered by oxidative phosphorylation, consisting of complexes that form the respiratory chain and ATP synthase. Researchers have examined the aerobic enzyme activities of these complexes and of enzymes supporting the Krebs cycle [e.g., succinate dehydrogenase, cytochrome c oxidase, citrate synthase (Gollnick et al., 1972; Costill et al., 1976; Saltin et al., 1977; Blomstrand et al., 1997)], which are highly interrelated (Larsen et al., 2012), as well as the mitochondrial volume density and location of mitochondria in locomotor muscles (Hoppeler et al., 1973; Ørtenblad et al., 2018). The total mitochondrial oxidative capacity can be assessed in permeabilised muscle fibers using high-resolution respirometer [i.e., oxidative phosphorylation rates, OXPHOS (Pesta and Gnaiger, 2012; Gnaiger and Group, 2020)]. Also, total mitochondrial oxidative capacity can be estimated for single muscle fibers using quantitative histochemistry of one of the aerobic enzymes: succinate dehydrogenase (SDH), an enzyme in the Krebs cycle, and complex II of the electron transport chain. Even though the enzyme is not rate limiting for the flux through the Krebs cycle (Blomstrand et al., 1997), SDH activity has shown to be proportional to the *ex vivo* $\dot{\text{V}}$O_2max_ of intact single muscle fibers and myocardial trabeculas in hyperoxia (van der Laarse et al., 1989; Des Tombe et al., 2002). Interestingly, both OXPHOS and SDH activity have also shown to be closely related to the $\dot{\text{V}}$O_2max_ of quadriceps muscles during one-legged exercise (Blomstrand et al., 1997; Jacobs and Lundby, 2021) and to body-mass-specific $\dot{\text{V}}$O_2max_ in humans (Costill et al., 1976; van der Zwaard et al., 2016a; Jacobs and Lundby, 2021). Recent observations in 68 cardiac patients, controls and elite cyclists demonstrated that mitochondrial oxidative capacity determined from SDH activity even scales proportionally with body-mass-specific $\dot{\text{V}}$O_2max_ during cycling, ranging 9.8–79.0 mL·kg^−1^·min^−1^ (van der Zwaard et al., 2016a). The $\dot{\text{V}}$O_2max_ measured during cycling was on average 90% of mitochondrial oxidative capacity (van der Zwaard et al., 2016a), indicating that oxygen supply limitations to the mitochondria are likely marginal at this high-intensity exercise.

### Capillarization

Muscle oxygen supply capacity to the muscle fibers is critical to sustain endurance performance. Endurance athletes are known for their well-developed capillarization compared to untrained or non-endurance athletes (Saltin et al., 1977; Sjøgaard, 1984; Tesch et al., 1984; Coyle et al., 1988; Aagaard et al., 2011; van der Zwaard et al., 2018a,b), demonstrating high number of capillaries around the fiber (~5–8), capillary-to-fiber ratios (~2.5–3.0), and capillary densities (~400–700 caps/mm^2^). Whereas, untrained individuals have 3–4 capillaries around the fiber (Saltin et al., 1977), professional road cyclists (Sjøgaard, 1984) and Olympic track cyclist (van der Zwaard et al., 2018b) displayed values as high as 9 capillaries around the fiber. High capillarization has been associated with a high $\dot{\text{V}}$O_2max_ (Saltin et al., 1977; Ingjer, 1978). Also, in competitive cycling, high capillary densities were strongly related to prolonged time-to-fatigue (Coyle et al., 1988). For cyclists displaying a similar lactate threshold, those with higher capillary densities demonstrated higher time-to-fatigue than those with lower capillary densities (Coyle et al., 1988). Importantly, capillarization may be a solution to the fiber type-fiber size paradox, given that cyclists with better developed capillarization were able to combine a larger FCSA with higher mitochondrial oxidative capacity (van der Zwaard et al., 2018b). Accordingly, rowers also possess large and highly oxidative muscle fibers, presumably because of their high capillary density (Larsson and Forsberg, 1980; Hagerman and Staron, 1983).

### Myoglobin

Not only capillarization, but also myoglobin contributes to muscle oxygen supply, as it transports oxygen within the muscle fibers. Literature on myoglobin content is scarce, especially in athletes. Myoglobin concentrations [Mb] have been reported in men and women obtained from homogenized muscle samples (Möller and Sylvén, 1981; Jansson and Sylvén, 1983). Alternatively, [Mb] could be determined in individual muscle fibers using quantitative histochemistry of biopsy sections (Lee-de Groot et al., 1998; van Beek-Harmsen et al., 2004), reflecting functional [Mb protein], and have been reported in cardiac patients and healthy controls (Bekedam et al., 2009), elite cyclists (van der Zwaard et al., 2018b), and elite hockey players (van der Zwaard et al., 2018a). [Mb] is typically ~50% higher in type-I compared to type-II muscle fibers (Jansson and Sylvén, 1983; Bekedam et al., 2009; van der Zwaard et al., 2018a), and this ratio does not seem to alter with training (van der Zwaard et al., 2018a). Of particular interest is the interaction of [Mb] and capillarization, as their combination (1) explained more variance in endurance cycling performance than capillarization alone (van der Zwaard et al., 2018b), and (2) explained which elite hockey players were able to combine a larger FCSA with higher mitochondrial oxidative capacity (*r* = 0.68; data from van der Zwaard et al., 2018a). These findings highlight the value of measuring both capillarization and functional [Mb] to characterize muscle fiber oxygen supply capacity.

### Muscle Glycogen Storage

Glycogen is an essential substrate for muscle metabolism during endurance exercise (Knuiman et al., 2015). It is well-known that glycogen depletion is strongly associated with muscular fatigue during endurance exercise (Ørtenblad et al., 2013; Alghannam et al., 2016), and endurance-trained individuals store substantially more glycogen in their muscles and can resynthesize glycogen faster (Piehl, 1974; Hickner et al., 1997). Therefore, endurance athletes benefit from a high glycogen availability, particularly in long-lasting endurance events. Interestingly, this has led to several nutritional-exercise strategies to manipulate muscle glycogen availability and subsequent cell signaling pathways regulating skeletal muscle adaptations (Burke et al., 2018).

## An Integrative Perspective On Endurance Performance

**Figure 2:**
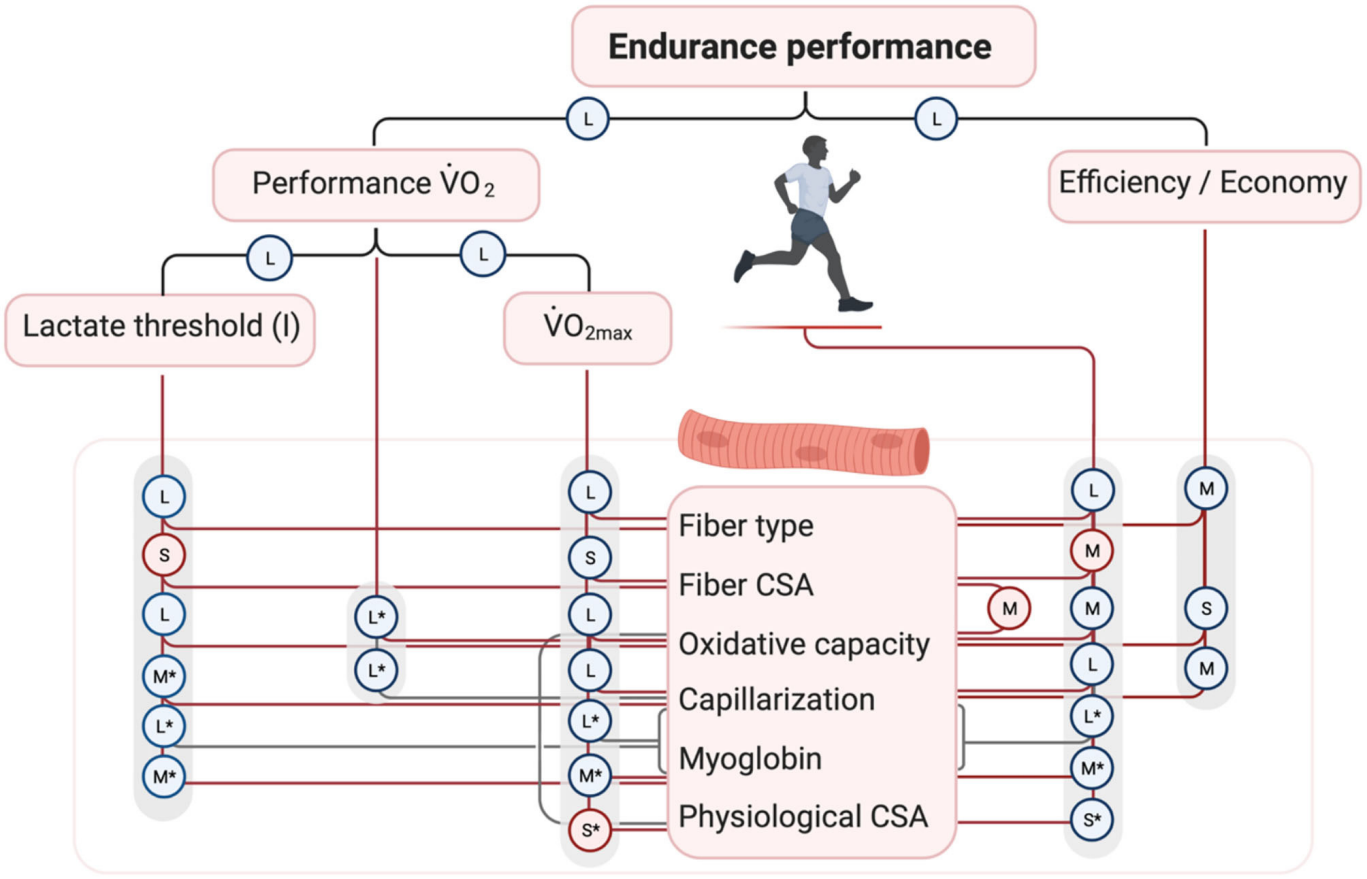
Overall schematic describing the underlying skeletal muscle determinants of endurance performance and whole-body determinants of endurance performance. The schematic advances on the theoretical framework by Joyner and Coyle (2008). Based on a meta-analysis of the existing literature, effect sizes of correlations between these determinants and endurance performance have been summarized in forest plots (see Supplementary Material). Random effect sizes were calculated based on Fisher's z transformation of correlations and with between-study variance estimations based on the “more conservative” Sidik-Jonkman estimator. Effect sizes were interpreted in accordance with Cohen (1992): negligible (*r* < 0.1), small (*r* = 0.1–0.29), medium (*r* = 0.3–0.5), and large (*r* > 0.5), and indicated with the symbols N, S, M, and L, respectively. Negative effects are shown in red circles, positive effects in blue circles. Black lines indicate effects of single whole-body determinants, red lines indicate effects of single muscle determinants and gray lines indicate integrative effects of multiple muscle determinants. *indicates that limited research is available (i.e., only 1 or 2 studies). Studies included in the meta-analysis:Hoppeler et al. (1973), Booth and Narahara (1974), Hultén et al. (1975), Costill et al. (1976), Bergh et al. (1978), Foster et al. (1978), Ingjer (1978), Rusko et al. (1978, 1980), Ivy et al. (1980a,b), Gregor et al. (1981), Inbar et al. (1981), Komi et al. (1981), Nygaard (1981), Zumstein et al. (1983), Coyle et al. (1984, 1988, 1992), Lortie et al. (1985), Neary et al. (1992, 2003), Farrell et al. (1993), Horowitz et al. (1994), Tonkonogi and Sahlin (1997), Dawson et al. (1998), Bishop et al. (2000), Passfield and Doust (2000), Hansen et al. (2002), Bentley and McNaughton (2003), Lucía et al. (2004), Mogensen et al. (2006), Baldari et al. (2007), Farina et al. (2007), Hansen and Sjøgaard (2007), Flynn et al. (2009), Iaia et al. (2009), Suriano et al. (2010), Jacobs et al. (2011), Kohn et al. (2011), Hopker et al. (2013), Jacobs and Lundby (2013), Støren et al. (2013), Hunter et al. (2015), Gifford et al. (2016), van der Zwaard et al. (2016a, 2018b), Vikmoen et al. (2016), Lundby et al. (2017), Cardinale et al. (2018), Dandanell et al. (2018), Mitchell et al. (2018), Batterson et al. (2020), Kovács et al. (2020).

We performed a meta-analysis (described in detail in Supplementary Material 1) to examine how skeletal muscle characteristics may contribute to endurance performance and whole-body determinants of endurance performance, of which the results are summarized in Figure 2. This figure displays reported relationships and their effect size from previous studies. Because no single determinant is able to explain all variance in endurance performance, statistical models have been established to include interactions between multiple macroscopic and microscopic determinants (whole-body and/or skeletal muscle, respectively). In line with the schematic of Joyner and Coyle (2008), the combination of performance $\dot{\text{V}}$O_2_ and gross efficiency could explain >90% of the variance in 1-h cycling in trained men (Hopker et al., 2013) and 15-km time trial in elite cyclists (data from van der Zwaard et al., 2018b). Subsequently, performance $\dot{\text{V}}$O_2_ was largely explained by a combination of the first lactate threshold and $\dot{\text{V}}$O_2max_ in these cyclists (86% explained variance; data from van der Zwaard et al., 2018b). Also, the combination of cycling efficiency, $\dot{\text{V}}$O_2max_ and the lactate threshold could explain >75% of the variance in 25-km cycling performance in trained men (Batterson et al., 2020). In professional cyclists, long-distance time-trial performance was highly correlated to the first lactate threshold (Lucía et al., 2004), and in competitive cyclists, a combination of the first lactate threshold with capillary density explained 92% of the differences in time-to-fatigue (Coyle et al., 1988), stressing the importance of oxidative capacity and oxygen delivery in regulating oxygen flux necessary to sustain performance. This narrative is supported by a study in highly-trained cyclists, demonstrating that 78% of the variance in 26-km time-trial could be explained by oxidative capacity of skeletal muscle, submaximal lactate concentration and ability to extract oxygen in skeletal muscle (Jacobs et al., 2011). Interestingly, recent observations in elite cyclists showed that 67% of the variance in performance $\dot{\text{V}}$O_2_ was explained by skeletal muscle determinants, which are a high muscle fiber oxidative capacity, high oxygen supply capacity ([Mb] × capillary-to-fiber ratio) and a small physiological cross-sectional area of the *M. vastus lateralis* (van der Zwaard et al., 2018b). While confirming the importance of oxygen supply and oxidative capacity, these results also reveal that muscle architecture [e.g., the physiological cross-sectional area (PCSA), the cross-sectional area of a muscle perpendicular to its muscle fibers], which can be obtained using non-invasive techniques, such as 3D ultrasound imaging (Weide et al., 2017), may add novel and valuable insights into endurance performance. At present, however, these whole-muscle characteristics have been largely overlooked as determinants of endurance performance. The finding that PCSA negatively contributed to performance $\dot{\text{V}}$O_2_ is in line with previous observations showing that a large FCSA may negatively affect endurance performance (Coyle et al., 1988; Bishop et al., 2000) and the lactate threshold (Bishop et al., 2000), likely because this increases the diffusion distance of oxygen to the mitochondria. Importantly, literature suggests that larger muscle FCSA or PCSA may (partly) be accommodated if the oxygen supply toward the muscle—capillarization, and myoglobin—is well-developed (van der Zwaard et al., 2018a,b). Here, non-invasive measurements with near-infrared spectroscopy may also provide valuable insights into spatial and temporal matching of oxygen supply and demand during exercise, and could explain a large proportion of the differences in endurance performance (Jacobs et al., 2011; van der Zwaard et al., 2016b; Batterson et al., 2020). In summary, integrative assessment of skeletal muscle determinants of endurance performance points toward optimisation of efficient type-I fibers with a high mitochondrial oxidative capacity and strongly encourages well-adjusted capillarization and myoglobin concentrations to accommodate the required oxygen flux during endurance performance, especially in a large muscle FCSA or PCSA with attenuated diffusion of oxygen to the mitochondria.

## Training-Induced Adaptations to Maximize Endurance Performance

Endurance training shifts velocity/power-duration relationships to the right, allowing athletes to perform a given distance faster or to sustain a given velocity longer (Jones and Carter, 2000). As such, endurance training plays a central role within exercise physiology (van der Zwaard et al., 2020), with training methods facilitating adaptations in macroscopic and microscopic physiological determinants in response to training intensity, duration, frequency, and periodization (Seiler, 2010; Hawley et al., 2018). For example, polarized training consisting predominantly of low-intensity, long-duration training along with some high-intensity training, may be optimal to enhance (whole-body determinants of) endurance performance (Stöggl and Sperlich, 2014; Tønnessen et al., 2014), through higher adaptive cellular signaling, gene expression, and stress responses (Seiler, 2010). Linking training behavior to molecular adaptations, Seiler (2010) previously suggested that mitochondrial biosynthesis might be driven through different pathways depending on exercise duration and intensity. These pathways include, but are not limited to, sustained contractile activity during long exercise bouts that induces chronic release of calcium, low energy status (low AMP:ATP ratio) with high-intensity exercise that increases AMPK and PGC-1α phosphorylation, and training-induced oxidative stress and altered redox states that affect PGC-1α, which trigger mitochondrial adaptations (Hood, 2001; Egan and Zierath, 2013). However, whether training intensity or volume is the most important to increase mitochondrial oxidative capacity remains highly debated (Bishop et al., 2019; MacInnis et al., 2019). Recent evidence suggests that too much high-intensity training may also adversely affect mitochondrial adaptations (Granata et al., 2020; Cardinale et al., 2021; Flockhart et al., 2021). Polarized training may therefore have an advantageous mix of low- and high-intensity exercise for maximizing mitochondrial adaptations.

To benefit from this enhanced oxidative capacity, oxygen supply needs to match oxygen demand (Hepple, 2000), which requires adaptations in capillary density and/or Mb content. High-intensity or (repeated-)sprint exercise may induce local tissue hypoxia (with reduced PO_2_) leading to stabilization of the transcription factor HIF-1α, which increases transcription of genes for capillary growth (Lundby et al., 2009; Egan and Zierath, 2013). Although controversies may exist (e.g., Millet and Brocherie, 2020; Siebenmann and Dempsey, 2020), when performed in hypoxia, high-intensity training also seems effective in increasing transcription of VEGF and Mb (Vogt et al., 2001; Kanatous et al., 2009; Faiss et al., 2013; Brocherie et al., 2018). Importantly, the prolonged low-intensity exercise in polarized training could stimulate muscle protein breakdown via enhanced expression of E3 ligases (Stefanetti et al., 2015), resulting in small muscle fibers that facilitate oxygen diffusion to the mitochondria. Growing evidence supports favorable adaptations (i.e., shift in muscle fiber phenotype (from less efficient myosin heavy chain IIX to more oxidative type myosins I and IIA), improvements in motor unit recruitment and firing frequency, greater muscle activation and intra- and inter-muscular coordination, greater muscle–tendon unit stiffness and biomechanical efficiency, changes in substrate utilization, metabolites accumulation, and important signaling cascades related to PGC-1α) following sprint interval training (Sloth et al., 2013), repeated-sprint training (Bishop et al., 2011; Girard et al., 2011; Taylor et al., 2015) or even resistance training (Blagrove et al., 2018) that are more and more implemented among (elite) endurance athletes. Note that training interventions require optimal periodisation, taking into account time windows of adaptation and decay of the underlying skeletal muscle determinants (Hickson and Rosenkoetter, 1981). Considering periodisation, dose-response relationships, and individual trainability, discovering what type of training is the most effective remains an unsolved question that nourish the endless debate among practitioners and researchers.

## Conclusion

Determinants of endurance performance have previously been established in the conceptual framework by Joyner and Coyle (2008). However, less is known on how the differences in endurance performance are explained by skeletal muscle characteristics and whole-body determinants. This review includes a meta-analysis that highlights the interrelationships and corresponding effect sizes between endurance performance, $\dot{\text{V}}$O_2max_, lactate threshold and efficiency/economy as well as skeletal muscle fiber type, fiber size, capillarization, myoglobin, mitochondrial oxidative capacity, and whole-muscle morphology. Integrative assessment of these determinants is encouraged, stressing the importance of capillarization and myoglobin, and training strategies to improve the physiological determinants have been mentioned, taking into account the different time windows of their adaptation and decay. In summary, optimisation of endurance performance requires careful design of training interventions that finetune modulation of exercise intensity, frequency and duration, and particularly periodisation with respect to the skeletal muscle determinants.

## Author Contributions

SZ, FB, and RJ conceived, designed the work, drafted, and revised the manuscript. SZ acquired, analyzed, and interpreted the data (from the meta-analysis). All authors read and approved the final version of the manuscript for publication.

## Conflict of Interest

The authors declare that the research was conducted in the absence of any commercial or financial relationships that could be construed as a potential conflict of interest.

## Publisher's Note

All claims expressed in this article are solely those of the authors and do not necessarily represent those of their affiliated organizations, or those of the publisher, the editors and the reviewers. Any product that may be evaluated in this article, or claim that may be made by its manufacturer, is not guaranteed or endorsed by the publisher.
