# Supplementary material for: Under the Hood: Skeletal Muscle Determinants of Endurance Performance
Source: Front Sports Act Living. 2021 Aug 4;3:719434. doi: 10.3389/fspor.2021.719434 (PMC8371266; doi:10.3389/fspor.2021.719434)
Supplement: Supplementary file 1 [file Data_Sheet_1.PDF]

Supplementary file 1:
Meta-analysis of skeletal muscle determinants of endurance performance

This supplementary file includes the meta-analysis that served as input for Figure 2 in the mini-review "*Under the hood: skeletal muscle determinants of endurance performance*".

Methodology:

In order to describe the relationships between skeletal muscle determinants and (whole-body determinants of) endurance performance in trained individuals, a literature search was performed using the PubMed database and the following search criteria:

((Endurance performance) OR (Time-to-exhaustion) OR (VO2max) OR (VO2peak) OR (Maximal oxygen uptake) OR (Maximal aerobic capacity) OR (Economy) OR (Efficiency) OR (Lactate threshold) OR (Ventilatory threshold) OR (Performance VO2)) AND (Skeletal muscle) AND ((Fiber type) OR (Fiber size) OR (FCSA) OR (Capillarization) OR (Capillary) OR (Myoglobin) OR (Mitochondria) OR (PCSA)).

A total number of 2,868 records were identified and screened for relevance, after which 2,546 records were excluded. From the remaining 322 studies, 3 records could not be retrieved and the other 319 records were assessed for eligibility based on article full-texts. Incorporation criteria include (i) population of trained or elite individuals and (ii) a reported coefficient of correlation between skeletal muscle determinants and (whole-body determinants of) endurance performance. Based on this, 32 articles were considered eligible and were included in the meta-analysis. An additional 22 items were included based on citation searching. The PRISMA flow diagram of the search has been presented below (Page et al., 2021).

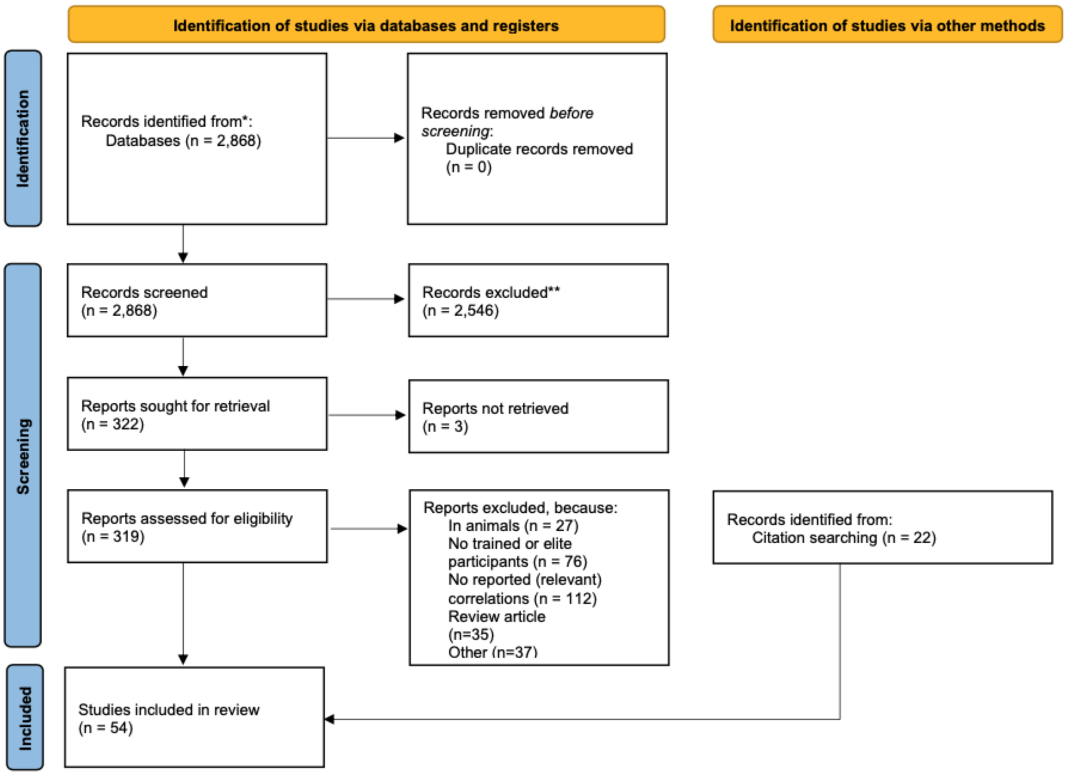

Figure 1. PRISMA flow diagram of the meta-analysis search and identification of eligible studies.

Based on the reported coefficients of correlation from these prior studies, random effect sizes were calculated based on Fisher's z transformation of correlations and with between-study variance estimations based on the ‘more conservative’ Sidik-Jonkman estimator. These effects were summarized in forest plots that are presented below, in which studies are referred to by their first author and year of publication. Average effects based on the random effects model were analysed and were used to construct Figure 2 within the mini review article. Effect sizes were interpreted in accordance with Cohen (1992): negligible (r<0.1), small (r=0.1-0.29), medium (r=0.3-0.5) and large (r>0.5). For some studies, additional descriptions were added (e.g. running / cycling or calf / thigh muscles) and the asterisk (*) indicates that coefficients of correlation were determined based on the data from the corresponding study. Please note that in line with the file-drawer problem, non-significant correlations have not always been reported in the analysed studies, and therefore observed effect sizes may be an overestimation of the true effect sizes (Rosenthal, 1979). Therefore, researchers are encouraged to report significant as well as non-significant correlation coefficients between skeletal muscle determinants and (whole body determinants of) endurance performance in future studies. All analysis were performed in R using the *meta* package.

Endurance performance:

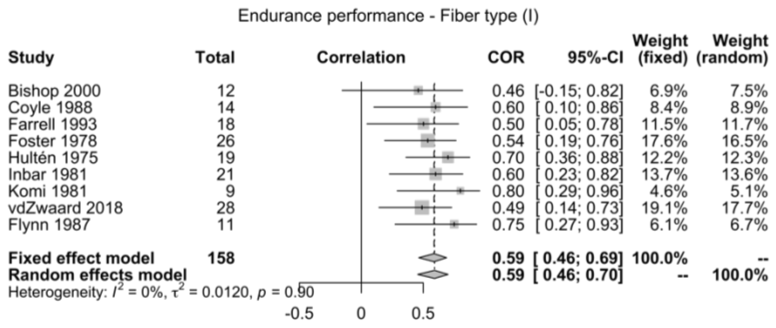

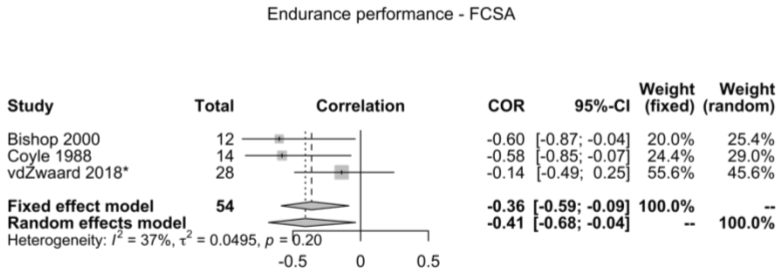

Endurance performance - Oxidative capacity

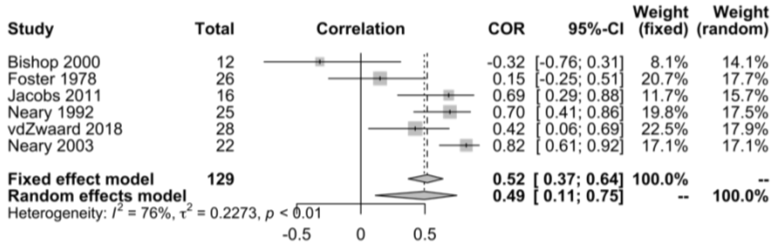

Endurance performance - Capillarization

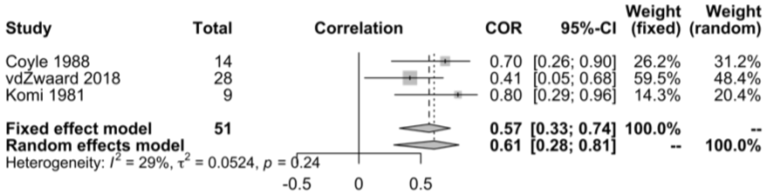

Endurance performance - Mb x capillarization

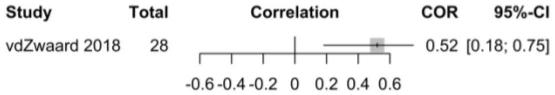

Endurance performance - Mb

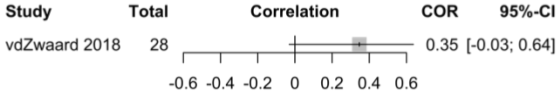

Endurance performance - PCSA

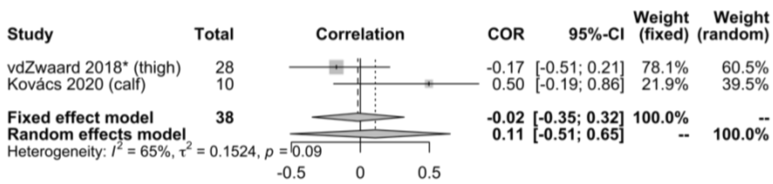

First lactate threshold

LT1 - Fiber type (I)

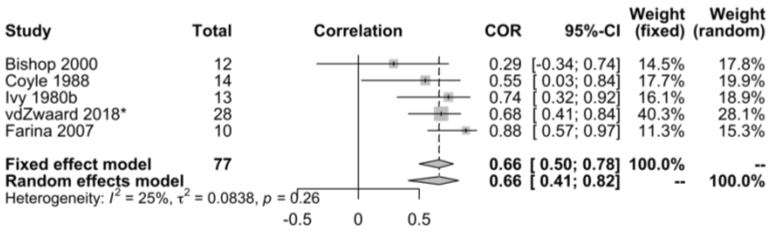

LT1 - FCSA

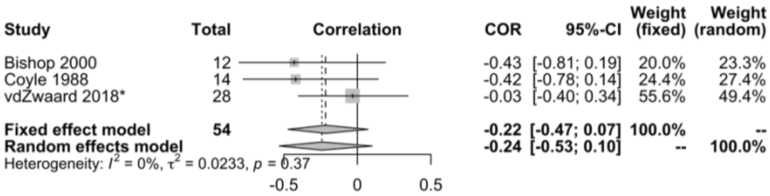

LT1 - Oxidative capacity

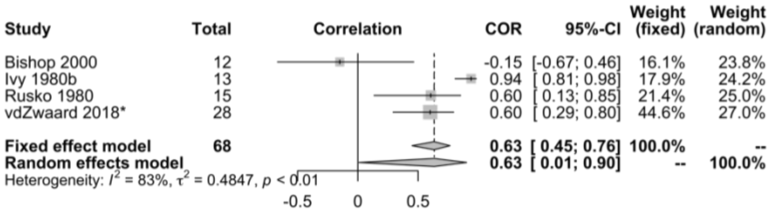

LT1 - Capillarization

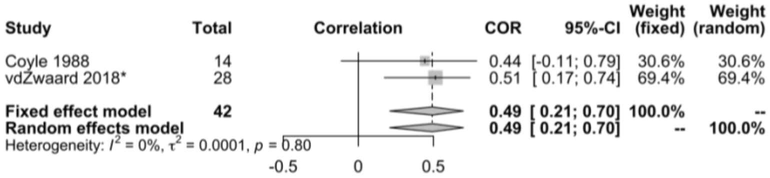

LT1 - Mb x capillarization

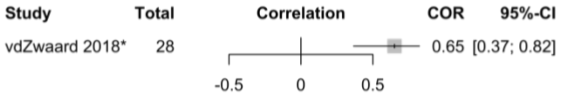

LT1 - Mb

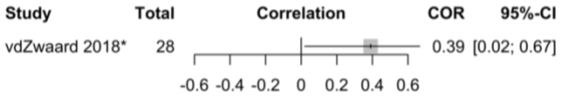

LT1 - PCSA

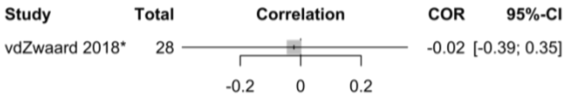

VO_{2max}

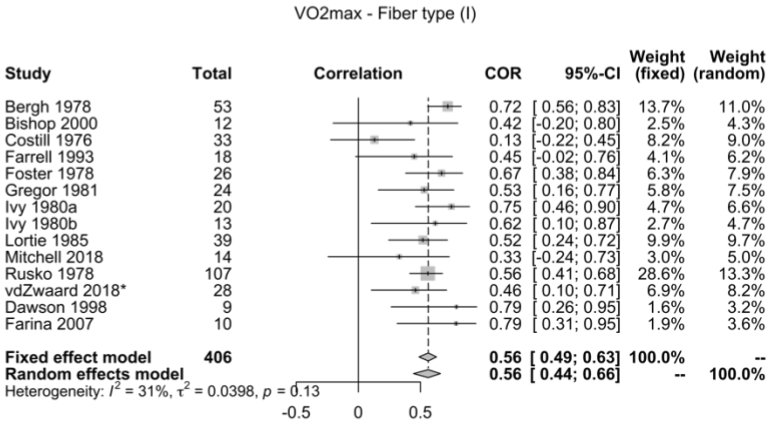
VO2max - Fiber type (I)
Study Total Correlation COR 95%-CI Weight (fixed) Weight (random)
Bergh 1978 53 0.72 [0.56; 0.83] 13.7% 11.0%
Bishop 2000 12 0.42 [-0.20; 0.80] 2.5% 4.3%
Costill 1976 33 0.13 [-0.22; 0.45] 8.2% 9.0%
Farrell 1993 18 0.45 [-0.02; 0.76] 4.1% 6.2%
Foster 1978 26 0.67 [0.38; 0.84] 6.3% 7.9%
Gregor 1981 24 0.53 [0.16; 0.77] 5.8% 7.5%
Ivy 1980a 20 0.75 [0.46; 0.90] 4.7% 6.6%
Ivy 1980b 13 0.62 [0.10; 0.87] 2.7% 4.7%
Lortie 1985 39 0.52 [0.24; 0.72] 9.9% 9.7%
Mitchell 2018 14 0.33 [-0.24; 0.73] 3.0% 5.0%
Rusko 1978 107 0.56 [0.41; 0.68] 28.6% 13.3%
vdZwaard 2018* 28 0.46 [0.10; 0.71] 6.9% 8.2%
Dawson 1998 9 0.79 [0.26; 0.95] 1.6% 3.2%
Farina 2007 10 0.79 [0.31; 0.95] 1.9% 3.6%
Fixed effect model 406 0.56 [0.49; 0.63] 100.0% --
Random effects model 0.56 [0.44; 0.66] -- 100.0%
Heterogeneity: I^2 = 31%, τ^2 = 0.0398, p = 0.13
-0.5 0 0.5

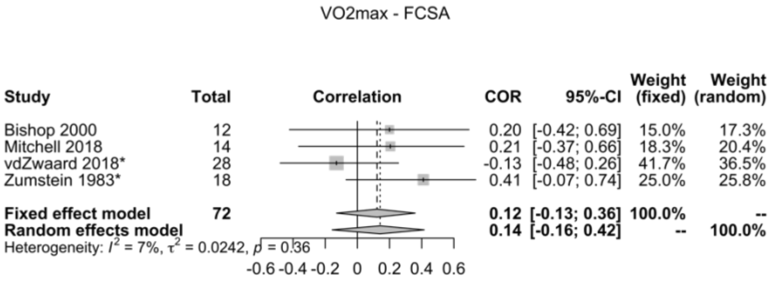
VO2max - FCSA
Study Total Correlation COR 95%-CI Weight (fixed) Weight (random)
Bishop 2000 12 0.20 [-0.42; 0.69] 15.0% 17.3%
Mitchell 2018 14 0.21 [-0.37; 0.66] 18.3% 20.4%
vdZwaard 2018* 28 -0.13 [-0.48; 0.26] 41.7% 36.5%
Zumstein 1983* 18 0.41 [-0.07; 0.74] 25.0% 25.8%
Fixed effect model 72 0.12 [-0.13; 0.36] 100.0% --
Random effects model 0.14 [-0.16; 0.42] -- 100.0%
Heterogeneity: I^2 = 7%, τ^2 = 0.0242, p = 0.36
-0.6 -0.4 -0.2 0 0.2 0.4 0.6

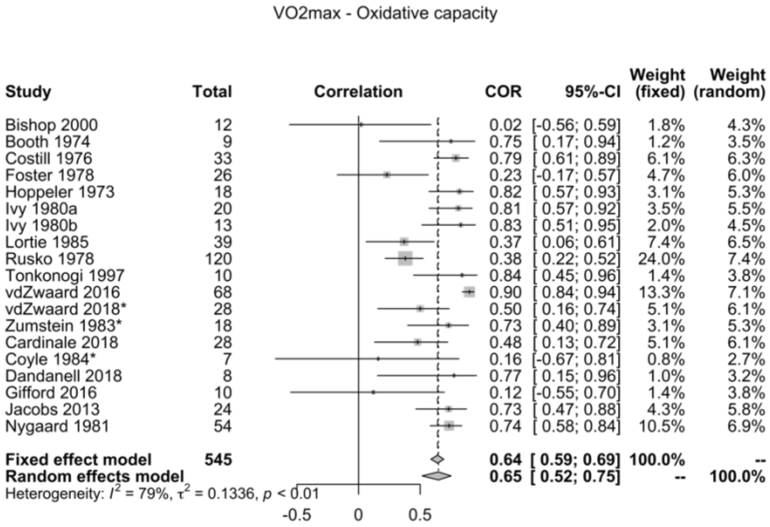
VO2max - Oxidative capacity
Study Total Correlation COR 95%-CI Weight (fixed) Weight (random)
Bishop 2000 12 0.02 [-0.56; 0.59] 1.8% 4.3%
Booth 1974 9 0.75 [0.17; 0.94] 1.2% 3.5%
Costill 1976 33 0.79 [0.61; 0.89] 6.1% 6.3%
Foster 1978 26 0.23 [-0.17; 0.57] 4.7% 6.0%
Hoppeler 1973 18 0.82 [0.57; 0.93] 3.1% 5.3%
Ivy 1980a 20 0.81 [0.57; 0.92] 3.5% 5.5%
Ivy 1980b 13 0.83 [0.51; 0.95] 2.0% 4.5%
Lortie 1985 39 0.37 [0.06; 0.61] 7.4% 6.5%
Rusko 1978 120 0.38 [0.22; 0.52] 24.0% 7.4%
Tonkonogi 1997 10 0.84 [0.45; 0.96] 1.4% 3.8%
vdZwaard 2016 68 0.90 [0.84; 0.94] 13.3% 7.1%
vdZwaard 2018* 28 0.50 [0.16; 0.74] 5.1% 6.1%
Zumstein 1983* 18 0.73 [0.40; 0.89] 3.1% 5.3%
Cardinale 2018 28 0.48 [0.13; 0.72] 5.1% 6.1%
Coyle 1984* 7 0.16 [-0.67; 0.81] 0.8% 2.7%
Dandanell 2018 8 0.77 [0.15; 0.96] 1.0% 3.2%
Gifford 2016 10 0.12 [-0.55; 0.70] 1.4% 3.8%
Jacobs 2013 24 0.73 [0.47; 0.88] 4.3% 5.8%
Nygaard 1981 54 0.74 [0.58; 0.84] 10.5% 6.9%
Fixed effect model 545 0.64 [0.59; 0.69] 100.0% --
Random effects model 0.65 [0.52; 0.75] -- 100.0%
Heterogeneity: I^2 = 79%, τ^2 = 0.1336, p < 0.01
-0.5 0 0.5

VO2max - Capillarization

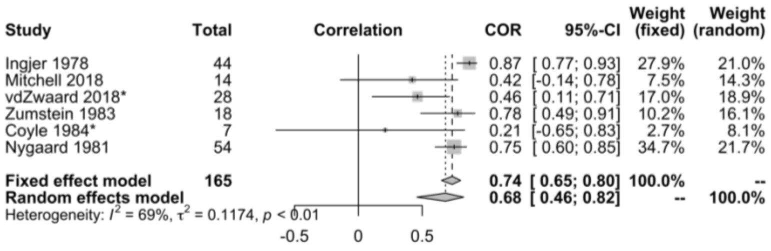

VO2max - Mb x capillarization

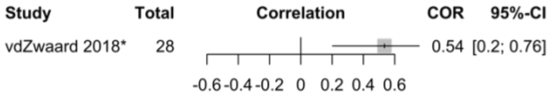

VO2max - Mb

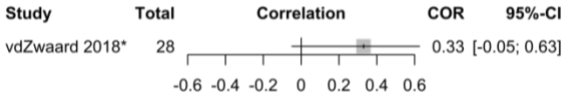

VO2max - PCSA

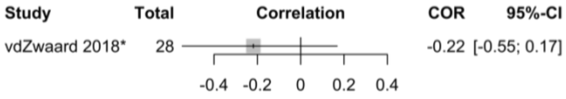

Efficiency

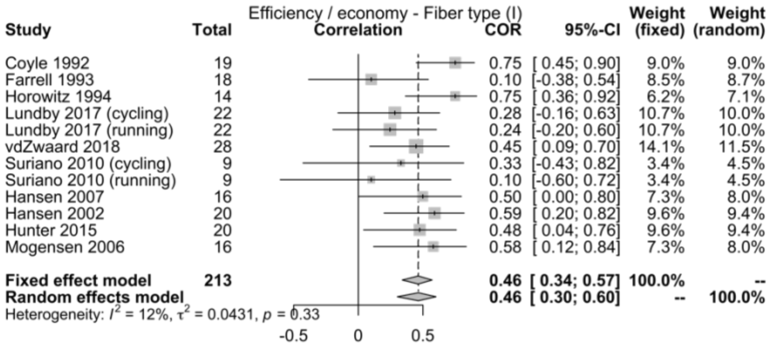
Efficiency / economy - Fiber type (I)
Study Total Correlation COR 95%-CI Weight (fixed) Weight (random)
Coyle 1992 19 0.75 [0.45; 0.90] 9.0% 9.0%
Farrell 1993 18 0.10 [-0.38; 0.54] 8.5% 8.7%
Horowitz 1994 14 0.75 [0.36; 0.92] 6.2% 7.1%
Lundby 2017 (cycling) 22 0.28 [-0.16; 0.63] 10.7% 10.0%
Lundby 2017 (running) 22 0.24 [-0.20; 0.60] 10.7% 10.0%
vdZwaard 2018 28 0.45 [0.09; 0.70] 14.1% 11.5%
Suriano 2010 (cycling) 9 0.33 [-0.43; 0.82] 3.4% 4.5%
Suriano 2010 (running) 9 0.10 [-0.60; 0.72] 3.4% 4.5%
Hansen 2007 16 0.50 [0.00; 0.80] 7.3% 8.0%
Hansen 2002 20 0.59 [0.20; 0.82] 9.6% 9.4%
Hunter 2015 20 0.48 [0.04; 0.76] 9.6% 9.4%
Mogensen 2006 16 0.58 [0.12; 0.84] 7.3% 8.0%
Fixed effect model 213 0.46 [0.34; 0.57] 100.0% --
Random effects model 0.46 [0.30; 0.60] -- 100.0%
Heterogeneity: I^2 = 12%, τ^2 = 0.0431, p = 0.33
-0.5 0 0.5

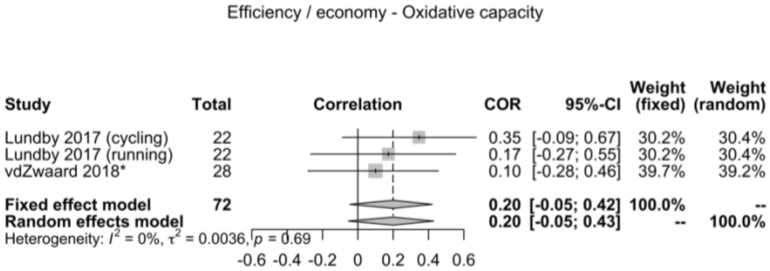
Efficiency / economy - Oxidative capacity
Study Total Correlation COR 95%-CI Weight (fixed) Weight (random)
Lundby 2017 (cycling) 22 0.35 [-0.09; 0.67] 30.2% 30.4%
Lundby 2017 (running) 22 0.17 [-0.27; 0.55] 30.2% 30.4%
vdZwaard 2018* 28 0.10 [-0.28; 0.46] 39.7% 39.2%
Fixed effect model 72 0.20 [-0.05; 0.42] 100.0% --
Random effects model 0.20 [-0.05; 0.43] -- 100.0%
Heterogeneity: I^2 = 0%, τ^2 = 0.0036, p = 0.69
-0.6 -0.4 -0.2 0 0.2 0.4 0.6

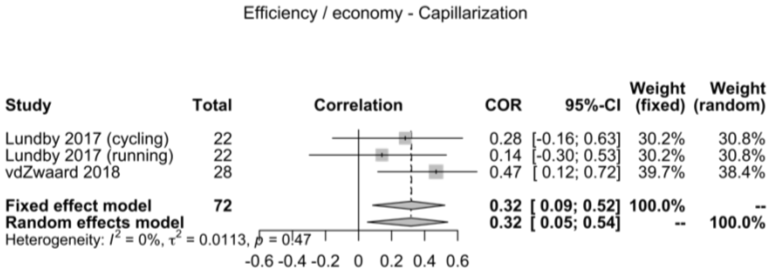
Efficiency / economy - Capillarization
Study Total Correlation COR 95%-CI Weight (fixed) Weight (random)
Lundby 2017 (cycling) 22 0.28 [-0.16; 0.63] 30.2% 30.8%
Lundby 2017 (running) 22 0.14 [-0.30; 0.53] 30.2% 30.8%
vdZwaard 2018 28 0.47 [0.12; 0.72] 39.7% 38.4%
Fixed effect model 72 0.32 [0.09; 0.52] 100.0% --
Random effects model 0.32 [0.05; 0.54] -- 100.0%
Heterogeneity: I^2 = 0%, τ^2 = 0.0113, p = 0.47
-0.6 -0.4 -0.2 0 0.2 0.4 0.6

Whole-body determinants of endurance performance

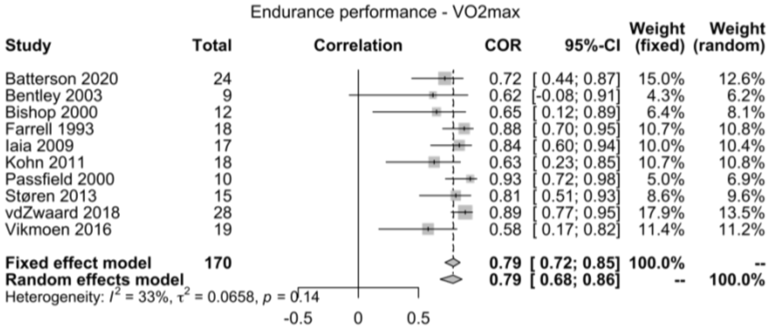

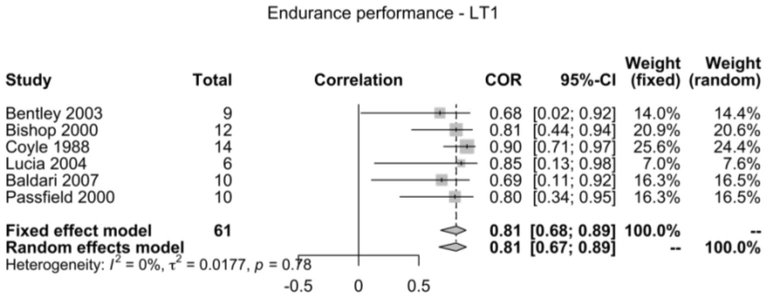

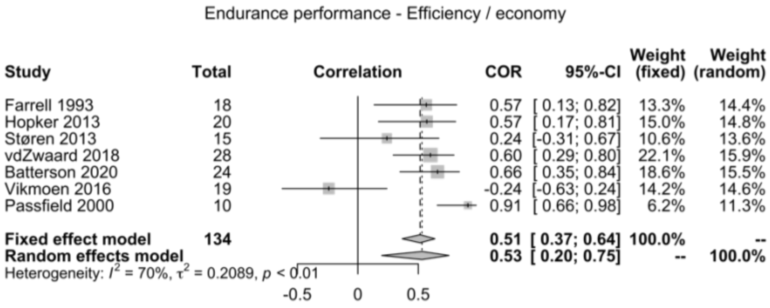

References

Baldari, C., Di Luigi, L., Silva, S. G. D., Gallotta, M. C., Emerenziani, G. P., Pesce, C., et al. (2007). Relationship between optimal lactate removal power output and Olympic triathlon performance. *J Strength Cond Res* 21, 1160–1165. doi:10.1519/R-21336.1.

Batterson, P. M., Norton, M. R., Hetz, S. E., Rohilla, S., Lindsay, K. G., Subudhi, A. W., et al. (2020). Improving biologic predictors of cycling endurance performance with near-infrared spectroscopy derived measures of skeletal muscle respiration: E pluribus unum. *Physiol Rep* 8, e14342. doi:10.14814/phy2.14342.

Bentley, D. J., and McNaughton, L. R. (2003). Comparison of W(peak), VO2(peak) and the ventilation threshold from two different incremental exercise tests: relationship to endurance performance. *J Sci Med Sport* 6, 422–435. doi:10.1016/s1440-2440(03)80268-2.

Bergh, U., Thorstensson, A., Sjödin, B., Hulten, B., Piehl, K., and Karlsson, J. (1978). Maximal oxygen uptake and muscle fiber types in trained and untrained humans. *Med Sci Sports* 10, 151–154.

Bishop, D., Jenkins, D. G., McEniery, M., and Carey, M. F. (2000). Relationship between plasma lactate parameters and muscle characteristics in female cyclists. *Med Sci Sports Exerc* 32, 1088–1093. doi:10.1097/00005768-200006000-00008.

Booth, F. W., and Narahara, K. A. (1974). Vastus lateralis cytochrome oxidase activity and its relationship to maximal oxygen consumption in man. *Pflugers Arch.* 349, 319–324. doi:10.1007/BF00588417.

Cardinale, D. A., Larsen, F. J., Schiffer, T. A., Morales-Alamo, D., Ekblom, B., Calbet, J. A. L., et al. (2018). Superior Intrinsic Mitochondrial Respiration in Women Than in Men. *Front Physiol* 9, 1133. doi:10.3389/fphys.2018.01133.

Costill, D. L., Daniels, J., Evans, W., Fink, W., Krahenbuhl, G., and Saltin, B. (1976). Skeletal muscle enzymes and fiber composition in male and female track athletes. *J Appl Physiol* 40, 149–154. doi:10.1152/jappl.1976.40.2.149.

Coyle, E. F., Coggan, A. R., Hopper, M. K., and Walters, T. J. (1988). Determinants of endurance in well-trained cyclists. *J Appl Physiol (1985)* 64, 2622–2630. doi:10.1152/jappl.1988.64.6.2622.

Coyle, E. F., Martin, W. H., Sinacore, D. R., Joyner, M. J., Hagberg, J. M., and Holloszy, J. O. (1984). Time course of loss of adaptations after stopping prolonged intense endurance training. *J Appl Physiol Respir Environ Exerc Physiol* 57, 1857–1864. doi:10.1152/jappl.1984.57.6.1857.

Coyle, E. F., Sidossis, L. S., Horowitz, J. F., and Beltz, J. D. (1992). Cycling efficiency is related to the percentage of type I muscle fibers. *Med Sci Sports Exerc* 24, 782–788.

Dandanell, S., Meinild-Lundby, A.-K., Andersen, A. B., Lang, P. F., Oberholzer, L., Keiser, S., et al. (2018). Determinants of maximal whole-body fat oxidation in elite cross-country skiers: Role of skeletal muscle mitochondria. *Scand J Med Sci Sports* 28, 2494–2504. doi:10.1111/sms.13298.

Dawson, B., Fitzsimons, M., Green, S., Goodman, C., Carey, M., and Cole, K. (1998). Changes in performance, muscle metabolites, enzymes and fibre types after short sprint training. *Eur J Appl Physiol Occup Physiol* 78, 163–169. doi:10.1007/s004210050402.

Farina, D., Ferguson, R. A., Macaluso, A., and De Vito, G. (2007). Correlation of average muscle fiber conduction velocity measured during cycling exercise with myosin heavy chain composition, lactate threshold, and VO2max. *J Electromyogr Kinesiol* 17, 393–400. doi:10.1016/j.jelekin.2006.03.003.

Farrell, P. A., Wilmore, J. H., Coyle, E. F., Billing, J. E., and Costill, D. L. (1993). Plasma lactate accumulation and distance running performance. *Medicine & Science in Sports & Exercise* 25, 1091–1097.

Flynn, M. G., Costill, D. L., Kirwan, J. P., Fink, W. J., and Dengel, D. R. (1987). Muscle fiber composition and respiratory capacity in triathletes. *Int J Sports Med* 8, 383–386. doi:10.1055/s-2008-1025690.

Foster, C., Costill, D. L., Daniels, J. T., and Fink, W. J. (1978). Skeletal muscle enzyme activity, fiber composition and VO2max in relation to distance running performance. *Europ. J. Appl. Physiol.* 39, 73–80. doi:10.1007/BF00421711.

Gifford, J. R., Garten, R. S., Nelson, A. D., Trinity, J. D., Layec, G., Witman, M. A. H., et al. (2016). Symmorphosis and skeletal muscle V̇O2 max : in vivo and in vitro measures reveal differing constraints in the exercise-trained and untrained human. *J Physiol* 594, 1741–1751. doi:10.1113/JP271229.

Gregor, R. J., Edgerton, V. R., Rozenek, R., and Castleman, K. R. (1981). Skeletal muscle properties and performance in elite female track athletes. *Europ. J. Appl. Physiol.* 47, 355–364. doi:10.1007/BF02332963.

Hansen, E. A., Andersen, J. L., Nielsen, J. S., and Sjøgaard, G. (2002). Muscle fibre type, efficiency, and mechanical optima affect freely chosen pedal rate during cycling. *Acta Physiol Scand* 176, 185–194. doi:10.1046/j.1365-201X.2002.01032.x.

Hansen, E. A., and Sjøgaard, G. (2007). Relationship between efficiency and pedal rate in cycling: significance of internal power and muscle fiber type composition. *Scand J Med Sci Sports* 17, 408–414. doi:10.1111/j.1600-0838.2006.00580.x.

Hopker, J. G., Coleman, D. A., Gregson, H. C., Jobson, S. A., Von der Haar, T., Wiles, J., et al. (2013). The influence of training status, age, and muscle fiber type on cycling efficiency and endurance performance. *Journal of Applied Physiology* 115, 723–729. doi:10.1152/japplphysiol.00361.2013.

Hoppeler, H., Lüthi, P., Claassen, H., Weibel, E. R., and Howald, H. (1973). The ultrastructure of the normal human skeletal muscle. A morphometric analysis on untrained men, women and well-trained orienteers. *Pflugers Arch* 344, 217–232. doi:10.1007/BF00588462.

Horowitz, J. F., Sidossis, L. S., and Coyle, E. F. (1994). High efficiency of type I muscle fibers improves performance. *Int J Sports Med* 15, 152–157. doi:10.1055/s-2007-1021038.

Hultén, B., Thorstensson, A., Sjödin, B., and Karlsson, J. (1975). Relationship between isometric endurance and fibre types in human leg muscles. *Acta Physiol Scand* 93, 135–138. doi:10.1111/j.1748-1716.1975.tb05799.x.

Hunter, G. R., McCarthy, J. P., Carter, S. J., Bamman, M. M., Gaddy, E. S., Fisher, G., et al. (2015). Muscle Fiber Type, Achilles Tendon Length, Potentiation, and Running Economy. *The Journal of Strength & Conditioning Research* 29, 1302–1309. doi:10.1519/JSC.0000000000000760.

Iaia, F. M., Hellsten, Y., Nielsen, J. J., Fernström, M., Sahlin, K., and Bangsbo, J. (2009). Four weeks of speed endurance training reduces energy expenditure during exercise and maintains muscle oxidative capacity despite a reduction in training volume. *J Appl Physiol (1985)* 106, 73–80. doi:10.1152/japplphysiol.90676.2008.

Inbar, O., Kaiser, P., and Tesch, P. (1981). Relationships between leg muscle fiber type distribution and leg exercise performance. *Int J Sports Med* 2, 154–159. doi:10.1055/s-2008-1034603.

Ingjer, F. (1978). Maximal aerobic power related to the capillary supply of the quadriceps femoris muscle in man. *Acta Physiol Scand* 104, 238–240. doi:10.1111/j.1748-1716.1978.tb06273.x.

Ivy, J. L., Costill, D. L., and Maxwell, B. D. (1980a). Skeletal muscle determinants of maximum aerobic power in man. *Eur J Appl Physiol Occup Physiol* 44, 1–8. doi:10.1007/BF00421757.

Ivy, J. L., Withers, R. T., Van Handel, P. J., Elger, D. H., and Costill, D. L. (1980b). Muscle respiratory capacity and fiber type as determinants of the lactate threshold. *J Appl Physiol Respir Environ Exerc Physiol* 48, 523–527. doi:10.1152/jappl.1980.48.3.523.

Jacobs, R. A., and Lundby, C. (2013). Mitochondria express enhanced quality as well as quantity in association with aerobic fitness across recreationally active individuals up to elite athletes. *J Appl Physiol (1985)* 114, 344–350. doi:10.1152/japplphysiol.01081.2012.

Jacobs, R. A., Rasmussen, P., Siebenmann, C., Díaz, V., Gassmann, M., Pesta, D., et al. (2011). Determinants of time trial performance and maximal incremental exercise in highly trained endurance athletes. *J Appl Physiol (1985)* 111, 1422–1430. doi:10.1152/japplphysiol.00625.2011.

Kohn, T. A., Essén-Gustavsson, B., and Myburgh, K. H. (2011). Specific muscle adaptations in type II fibers after high-intensity interval training of well-trained runners. *Scand J Med Sci Sports* 21, 765–772. doi:10.1111/j.1600-0838.2010.01136.x.

Komi, P. V., Ito, A., Sjödin, B., Wallenstein, R., and Karlsson, J. (1981). Muscle metabolism, lactate breaking point, and biomechanical features of endurance running. *Int J Sports Med* 2, 148–153. doi:10.1055/s-2008-1034602.

Kovács, B., Kóbor, I., Gyimes, Z., Sebestyén, Ö., and Tihanyi, J. (2020). Lower leg muscle-tendon unit characteristics are related to marathon running performance. *Sci Rep* 10, 17870. doi:10.1038/s41598-020-73742-5.

Lortie, G., Simoneau, J. A., Hamel, P., Boulay, M. R., and Bouchard, C. (1985). Relationships between skeletal muscle characteristics and aerobic performance in sedentary and active subjects. *Eur J Appl Physiol Occup Physiol* 54, 471–475. doi:10.1007/BF00422954.

Lucía, A., Hoyos, J., Pérez, M., Santalla, A., Earnest, C. P., and Chicharro, J. L. (2004). Which laboratory variable is related with time trial performance time in the Tour de France? *British Journal of Sports Medicine* 38, 636–640. doi:10.1136/bjsm.2003.008490.

Lundby, C., Montero, D., Gehrig, S., Andersson Hall, U., Kaiser, P., Boushel, R., et al. (2017). Physiological, biochemical, anthropometric, and biomechanical influences on exercise economy in humans. *Scand J Med Sci Sports* 27, 1627–1637. doi:10.1111/sms.12849.

Mitchell, E. A., Martin, N. R. W., Bailey, S. J., and Ferguson, R. A. (2018). Critical power is positively related to skeletal muscle capillarity and type I muscle fibers in endurance-trained individuals. *J Appl Physiol (1985)* 125, 737–745. doi:10.1152/japplphysiol.01126.2017.

Mogensen, M., Bagger, M., Pedersen, P. K., Fernström, M., and Sahlin, K. (2006). Cycling efficiency in humans is related to low UCP3 content and to type I fibres but not to mitochondrial efficiency. *J Physiol* 571, 669–681. doi:10.1113/jphysiol.2005.101691.

Neary, J. P., Martin, T. P., and Quinney, H. A. (2003). Effects of taper on endurance cycling capacity and single muscle fiber properties. *Med Sci Sports Exerc* 35, 1875–1881. doi:10.1249/01.MSS.0000093617.28237.20.

Neary, J. P., Martin, T. P., Reid, D. C., Burnham, R., and Quinney, H. A. (1992). The effects of a reduced exercise duration taper programme on performance and muscle enzymes of endurance cyclists. *Eur J Appl Physiol Occup Physiol* 65, 30–36. doi:10.1007/BF01466271.

Nygaard, E. (1981). Skeletal muscle fibre characteristics in young women. *Acta Physiol Scand* 112, 299–304. doi:10.1111/j.1748-1716.1981.tb06820.x.

Page, M. J., McKenzie, J. E., Bossuyt, P. M., Boutron, I., Hoffmann, T. C., Mulrow, C. D., et al. (2021). The PRISMA 2020 statement: an updated guideline for reporting systematic reviews. *BMJ* 372, n71. doi:10.1136/bmj.n71.

Passfield, L., and Doust, J. H. (2000). Changes in cycling efficiency and performance after endurance exercise. *Med Sci Sports Exerc* 32, 1935–1941. doi:10.1097/00005768-200011000-00018.

Rosenthal, R. (1979). The file drawer problem and tolerance for null results. *Psychological Bulletin* 86, 638–641. doi:10.1037/0033-2909.86.3.638.

Rusko, H., Havu, M., and Karvinen, E. (1978). Aerobic performance capacity in athletes. *Eur J Appl Physiol Occup Physiol* 38, 151–159. doi:10.1007/BF00421531.

Rusko, H., Rahkila, P., and Karvinen, E. (1980). Anaerobic threshold, skeletal muscle enzymes and fiber composition in young female cross-country skiers. *Acta Physiol Scand* 108, 263–268. doi:10.1111/j.1748-1716.1980.tb06532.x.

Støren, Ø., Ulevåg, K., Larsen, M. H., Støa, E. M., and Helgerud, J. (2013). Physiological determinants of the cycling time trial. *The Journal of Strength & Conditioning Research* 27, 2366–2373. doi:10.1519/JSC.0b013e31827f5427.

Suriano, R., Edge, J., and Bishop, D. (2010). Effects of cycle strategy and fibre composition on muscle glycogen depletion pattern and subsequent running economy. *Br J Sports Med* 44, 443–448. doi:10.1136/bjsm.2007.046029.

Tonkonogi, M., and Sahlin, K. (1997). Rate of oxidative phosphorylation in isolated mitochondria from human skeletal muscle: effect of training status. *Acta Physiol Scand* 161, 345–353. doi:10.1046/j.1365-201X.1997.00222.x.

van der Zwaard, S., de Ruiter, C. J., Noordhof, D. A., Sterrenburg, R., Bloemers, F. W., de Koning, J. J., et al. (2016). Maximal oxygen uptake is proportional to muscle fiber oxidative capacity, from chronic heart failure patients to professional cyclists. *J Appl Physiol (1985)* 121, 636–645. doi:10.1152/japplphysiol.00355.2016.

van der Zwaard, S., van der Laarse, W. J., Weide, G., Bloemers, F. W., Hofmijster, M. J., Levels, K., et al. (2018). Critical determinants of combined sprint and endurance performance: an integrative analysis from muscle fiber to the human body. *FASEB J.* 32, 2110–2123. doi:10.1096/fj.201700827R.

Vikmoen, O., Raastad, T., Seynnes, O., Bergstrøm, K., Ellefsen, S., and Rønnestad, B. R. (2016). Effects of Heavy Strength Training on Running Performance and Determinants of Running Performance in Female Endurance Athletes. *PLoS One* 11, e0150799. doi:10.1371/journal.pone.0150799.

Zumstein, A., Mathieu, O., Howald, H., and Hoppeler, H. (1983). Morphometric analysis of the capillary supply in skeletal muscles of trained and untrained subjects--its limitations in muscle biopsies. *Pflugers Arch* 397, 277–283. doi:10.1007/BF00580261.
